# Transcriptional Profiling of Human Liver Identifies Sex-Biased Genes Associated with Polygenic Dyslipidemia and Coronary Artery Disease

**DOI:** 10.1371/journal.pone.0023506

**Published:** 2011-08-12

**Authors:** Yijing Zhang, Kathrin Klein, Aarathi Sugathan, Najlla Nassery, Alan Dombkowski, Ulrich M. Zanger, David J. Waxman

**Affiliations:** 1 Division of Cell and Molecular Biology, Department of Biology, Boston University, Boston, Massachusetts, United States of America; 2 Dr. Margarete Fischer-Bosch Institute of Clinical Pharmacology, Stuttgart, Germany; 3 Division of Clinical Pharmacology and Toxicology, Department of Pediatrics, Wayne State University, Detroit, Michigan, United States of America; National Cancer Institute, United States of America

## Abstract

Sex-differences in human liver gene expression were characterized on a genome-wide scale using a large liver sample collection, allowing for detection of small expression differences with high statistical power. 1,249 sex-biased genes were identified, 70% showing higher expression in females. Chromosomal bias was apparent, with female-biased genes enriched on chrX and male-biased genes enriched on chrY and chr19, where 11 male-biased zinc-finger KRAB-repressor domain genes are distributed in six clusters. Top biological functions and diseases significantly enriched in sex-biased genes include transcription, chromatin organization and modification, sexual reproduction, lipid metabolism and cardiovascular disease. Notably, sex-biased genes are enriched at loci associated with polygenic dyslipidemia and coronary artery disease in genome-wide association studies. Moreover, of the 8 sex-biased genes at these loci, 4 have been directly linked to monogenic disorders of lipid metabolism and show an expression profile in females (elevated expression of *ABCA1*, *APOA5* and *LDLR*; reduced expression of *LIPC*) that is consistent with the lower female risk of coronary artery disease. Female-biased expression was also observed for *CYP7A1*, which is activated by drugs used to treat hypercholesterolemia. Several sex-biased drug-metabolizing enzyme genes were identified, including members of the *CYP*, *UGT*, *GPX* and *ALDH* families. Half of 879 mouse orthologs, including many genes of lipid metabolism and homeostasis, show growth hormone-regulated sex-biased expression in mouse liver, suggesting growth hormone might play a similar regulatory role in human liver. Finally, the evolutionary rate of protein coding regions for human-mouse orthologs, revealed by dN/dS ratio, is significantly higher for genes showing the same sex-bias in both species than for non-sex-biased genes. These findings establish that human hepatic sex differences are widespread and affect diverse cell metabolic processes, and may help explain sex differences in lipid profiles associated with sex differential risk of coronary artery disease.

## Introduction

Mammalian sex determination is initiated by the *SRY* gene, which activates a developmental pathway leading to testis formation and establishes life-long sex differences in the patterns of gonadal hormone secretion [1]. Gonadal hormones, in turn, exert permanent differentiating effects (‘organizational’ actions) as well as short-term stimulatory effects that lead to sex differences in gene expression in multiple tissues [2]. Sex differences are also induced by non-gonadal signals and factors, including direct sex-biased effects of individual X and Y-chromosome genes [3]. Epigenetic modifications also play an important role in the development and maintenance of sexual dimorphism [4] by processes such as genetic imprinting [5], [6] and X-chromosome inactivation [7], [8], [9]. Sex differences characterize histone acetylation and histone methylation [10] and the expression of certain histone demethylases [11], [12]. Sexual differentiation is thus achieved through a complex interplay of multiple mechanisms [13].

Global gene expression studies in mouse and rat liver have identified >1,000 sex-dependent transcripts, which collectively have a major impact on hepatic physiology, inflammatory responses, diseased states, and the metabolism of steroids, drugs and environmental chemicals [14], [15], [16]. However, very little is known about the sex-dependence of gene expression in human liver. Small but pharmacologically significant sex differences in the expression of certain human hepatic drug-metabolizing CYP enzymes have been reported, most notably for CYP3A4 [17], [18], however, only limited efforts have been made to identify sex differences in human liver on a larger scale [19]. Such studies have the potential to elucidate clinically important sex differences in human hepatic physiology and pathophysiology, including sex differences in circulating lipid profiles, which are more favorable in women [20], [21] and are associated with their lower risk of cardiovascular disease compared to men [22], [23]. Recent genome wide association studies (GWAS) identified 22 loci associated with sex-biased serum lipid phenotypes [24], however, it is not known whether sex differences characterize gene expression from these or other loci contributing to lipid metabolism.

The present study was undertaken to characterize sex differences in human liver on a genome-wide scale using a large liver sample collection, which allows for detection of small expression differences with high statistical power. Using this approach, we identify 1,249 genes that show significant sex differences in expression, 70% of which are more highly expressed in females. We show that hepatic sex-biased genes are enriched in functions related to transcription, chromosome organization and sexual reproduction, among others. Furthermore, we report that sex-biased gene expression is most significantly associated with genes that participate in or regulate lipid metabolism, several of which have previously been associated with polygenic dyslipidemia and cardiovascular disease in GWAS analyses or are established drug targets for treatment of hyperlipidemia and hypercholesterolemia. We also report that half of the mouse orthologs of sex-biased human hepatic genes, in particular those involved in lipid metabolism and homeostasis, show sex-biased expression in mouse liver, where growth hormone (GH) is the major regulator of sex differences, and that genes that show the same sex bias in human and mouse liver have evolved more rapidly than non-sex-biased genes. These latter findings provide insight into species similarities, as well as species differences, in liver sex specificity.

## Methods

### Ethics Statement

The study was approved by the ethics committees of the medical faculties of the Charité, Humboldt University Berlin and the University of Tuebingen, and the institutional review board of Boston University, and was conducted in accordance with the Declaration of Helsinki. Written informed consent was obtained from each patient.

### Human liver panel

Human liver samples (112 male samples, 112 female samples; German residents of white ethnicity) were obtained from patients undergoing liver surgery at the Department of General, Visceral and Transplantation Surgery (Nuessler AK and Neuhaus P, Campus Virchow, University Medical Center Charité, Humbold University in Berlin, Germany). The average age of the subjects was 55.8±13.3 yr (males) and 55.5±14.7 yr (females) (Table S1A). Subjects had undergone surgery to have primary or metastatic liver tumors removed, or had hepatic tissue resected for other reasons. Only non-tumorous liver tissue was collected, and the absence of tumorous material was confirmed for all samples by histochemical analysis. Livers from donors with severe liver disease (viral hepatitis, human immunodeficiency virus, cirrhosis) or chronic alcoholism were excluded from the study. For additional information see Table S1A. Additional information is provided elsewhere [25] for 150 of the livers used in the present study. Liver tissue was stored at −80°C.

### RNA processing and microarray analysis

RNA was isolated from liver tissue by Trizol extraction and Qiagen RNeasy-Mini Kit with on-column DNase treatment [26]. Only high quality RNA preparations were used in this study, as determined by Agilent Bioanalyzer (RIN value >7 using Agilent Nano-Lab Chip Kit; Agilent Technologies, Waldbronn, Germany). A first set of randomized liver RNA pools was generated by randomly distributing the 112 male and 112 female liver RNA samples into 8 pools comprised of 14 male liver samples each (pools M1 to M8) and 8 pools of 14 female liver samples each (pools F1 to F8). Each pool was prepared by combining 0.5 µg total RNA from each of the 14 livers in the pool, to give 7 µg RNA in a final volume of 30 µl. The final RNA concentration was determined by Nanodrop analysis (Thermo Fisher Scientific Inc., Waltham, MA). A second set of 16 pools was prepared from the same set of 224 liver samples in the same way (male pools M9 to M16 and female pools F9 to F16) (Table S1B).

The 16 liver RNA pools of each sex were used in a total of 16 two-color, male vs. female hybridization microarrays by pairing pool M1 with pool F1, pool M2 with pool F2, etc. Fluorescent labeling of RNA and hybridization of the Alexa 555-labeled and Alexa 647-labeled amplified RNA samples to Agilent Whole Human Genome oligonucleotide microarrays (4×44K format; Agilent Technology, Palo Alto, CA; catalog # G4112F) was carried out, with dye swaps to eliminate dye bias [27], [28]. TIFF images of each scanned slide were analyzed using Agilent's feature extraction software followed by linear and LOWESS normalization and initial data analysis using Rosetta Resolver (version 5.1, Rosetta Biosoftware, Seattle, WA) [29]. The full set of normalized expression ratios and *p*-values is available at the Gene Expression Omnibus web site (http://www.ncbi.nlm.nih.gov/geo) as GEO series GSE23766.

### Microarray annotation and data analysis

41,000 probe sequences (60 nt long) provided by Agilent were mapped to the hg19 human genome using BLAT [30]. Probes with at least 56 nt sequence overlap with the genomic coordinates of hg19 or with GeneBank mRNAs were assigned the indicated annotation. Where available, RefSeq annotations were assigned to each probe as the highest priority, followed by non-RefSeq mRNA, then Ensembl, spliced EST, and finally unspliced ESTs annotations in order of decreasing priority. When two or more probes mapping to the same gene name showed the same sex-bias, only the probe with the highest composite array score (defined below) was retained. Probes associated with the same gene name but different sex-bias were retained, resulting in 33,250 non-redundant probes.

The fold-change was defined as the normalized male/female expression ratio for ratios >1, and as the negative inverse of the normalized male/female expression ratio for ratios <1. For each probe, a mean fold-change and *p*-value was calculated based on the set of 16 microarray expression ratios using the Rosetta Resolver-based error model [29]. The error model uses technology-specific data parameters to stabilize intensity variation estimates, along with error-weighted averaging of replicates. This approach has been demonstrated to provide an effective increase in statistical power [29]. In the present study, the mean standard deviation of log_10_ ratios for the 16 replicate arrays was 0.0896; power analysis based on this variance indicated that a fold change of 1.17 can be detected with a power of 0.8 (80%). A composite array score, ranging from 8–16, was also determined based on the number of arrays out of 16 that showed agreement with respect to whether the corresponding gene (i.e., transcript) was expressed at a higher level in the male liver pool (fold-change>1; male-biased expression) or at a higher level in the female liver pool (fold-change<−1; female-biased expression). A total of 1,249 probes (genes), listed in Table S2A, showed sex-biased expression with high stringency based on a combination of the following three criteria: mean |fold change| between male and female liver >1.15, *p*-value<0.005, and composite array score ≥14. This list of 1,249 sex-biased genes eliminates 13 probes that did not match any genomic region or that matched >3 sites across the genome but could not be mapped to any GeneBank mRNAs. 33 other probes met the |fold change| and the composite array score criteria but not the *p*-value threshold and were excluded, to eliminate probes with high variance in fold-change.

An apparent false discovery rate (FDR) was calculated as follows: of the 33,250 non-redundant probes, 4,734 had a mean |fold change|>1.15. The number of probes expected to meet the fold-change and composite array score *k*≥14 by chance is:
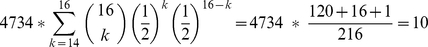
The actual number of probes that passed the combination threshold was 1,295, corresponding to an apparent FDR of 10/1295, or 0.77%. In an alternative approach to calculating the FDR, a *p*-value<0.005 was applied to the 4,734 probes that exhibited a |fold change|>1.15, resulting in 4,734 * 0.005 = 24 probes expected to meet the combined criteria, whereas 2,575 probes actually passed this combined threshold, corresponding to an apparent FDR of 24/2575 = 0.93%. Finally, given our rigorous experimental design and the Rosetta error model, the FDR remains very low even when applying statistical tests to the entire probe set. Thus, applying a *p*-value cutoff of 0.005 to all 41,000 probes on the microarray, 6,902 probes were identified as showing statistically significant sex differences, as compared to 41,000 * 0.005 = 205 probes that are expected to be identified by chance, corresponding to an FDR of 205/6,902 = 3%.

Where indicated, the composite array score filter was relaxed to ≥13 (1,855 probes) or to ≥12 (2,303 probes) to test the robustness of conclusions drawn from the most stringent cutoff (composite array score ≥14, |fold change|>1.15 and *p*<0.005). Table S2A and Table S2B present the gene lists, fold-changes, *p*-values and composite scores at all three levels of significance. Chi-square test was used to test the significance of apparent differences in sex-biased gene distributions across chromosomes. These analyses were carried out using sets of sex-biased genes determined at three levels of significance, namely, composite array score >14, 13 and 12, respectively, all combined with *p*-value<0.005 and |fold-change|>1.15. Only those genes whose microarray probes showed a single hit across the genome based on BLAT analysis [30] were used for chromosome mapping analysis, including analysis of the distribution of sex-biased genes on chromosome 19 and the sex-chromosomes.

Hierarchical clustering and heat map generation were carried out using Cluster [31] and Java Treeview [32], respectively. Enrichment of Gene Ontology, protein domain, pathway, and functional categories was determined using DAVID (http://david.abcc.ncifcrf.gov). The Core Analysis function of Ingenuity Pathway Analysis (Ingenuity System Inc, USA) was used to identify biological functions, pathways and networks associated with the 1,249 sex-biased genes. To examine the sex-dependent expression of genes related to ADME (absorption, distribution, metabolism and excretion), we used a list of 300 ADME genes (http://pharmaadme.org/) supplemented by 71 ADME-related genes from http://pharmaadme.org/ and 42 genes comprised of members of the *CYP*, *FMO*, *UGT*, *SULT*, *GST*, *NAT*, *ADH*, *ALDH* and *ARS* gene families not represented in the 300 ADME or 71 ADME-related lists.

### Comparison to 465 human liver microarray dataset

Validation of microarray results was carried out using an independent cohort of 465 human livers (253 males and 212 females) based on microarray analysis and annotations reported by Schadt et al. [33] with expression data and sex identifiers downloaded from http://sage.fhcrc.org/downloads/downloads.php. The dataset represents mostly post-mortem liver samples, primarily from Caucasian individuals, who were prospective organ donors and were obtained from three independent tissue resource centers, at Vanderbilt University, University of Pittsburgh, and Merck Research Laboratories. This 465 liver dataset includes 757 RefSeq genes in common with the 1,019 sex-biased RefSeq genes identified in this study based on our 224 liver dataset (Table S2). The ratio of average male expression to average female expression was calculated across the full set of 465 liver samples for all 757 genes, and a two-tail t-test *p*-value was determined for the resultant set of male to female expression ratios. For genes represented by duplicate microarray probes, only the probe with the lowest *p*-value was kept. Four of the 757 genes (*DAZ2*, *ZFY*, *DDX3Y/DDX3X*, *LRRC6*) showed large differences in sex ratio between the two studies and were excluded from further analysis, leaving 753 genes; these include 195 male-biased genes and 558 female-biased genes. *DAZ2* and *ZFY* are Y-chromosome genes that showed the expected high male/female ratio in our array dataset (ratios of 14 and 59, respectively) but not in the 465 liver dataset (male/female ratios of 1.2 and 0.53, respectively) suggesting cross hybridization to non-Y chromosome sequences. *DDX3Y/DDX3X* showed strong (16-fold) male-biased expression in the 465 liver dataset, but only 1.66-fold male bias in our dataset, while *LRRC6*, characterized as a testis-specific gene, showed 1.22-fold male-biased expression in our dataset, but strong (4.0-fold) female-biased expression in the 465 liver dataset. Gene Set Enrichment Analysis (GSEA; www.broadinstitute.org/gsea/) was used to compare the overall profile of sex-biased genes in our 224 liver dataset to that of the 465 liver dataset. 20,415 of the 40,638 microarray probes in the 465 liver dataset have RefSeq gene symbols in common with our microarray platform; 7,061 of these probes represent duplicated gene symbols and were removed, leaving 13,324 probes (probes having the lowest male/female t-test-based *p*-value were retained). Expression data for all 465 livers for the 13,354 RefSeq probes was used as input for GSEA and compared to the above set of 195 male-biased genes, and separately, to the set of 558 female-biased genes identified from the 224 liver dataset.

Pearson correlation was calculated between the sets of male/female expression log2-ratios determined for our 224 liver dataset and the 465 liver set. Permutation analysis was used to determine the significance of the correlation coefficient between each dataset, as follows. 16 male and 16 female liver samples (equal to the number of liver sample pools analyzed on our arrays) were randomly selected from the set of 465 liver samples, and male/female expression ratios were calculated for the 753 common genes specified above. A second male/female ratio was calculated for the remaining 433 liver samples. The Pearson correlation coefficient was recorded, and the procedure was repeated 1,000 times, giving 1,000 correlation coefficients. In other analyses, male/female expression ratios were calculated for subsets of the 465 liver set, comprised of individuals aged 15–52 (216 liver samples) and individuals aged 58 and older (165 liver samples). A Pearson correlation coefficient of 0.52 was calculated for the two sets of log2-ratios of the two age-determined liver subsets; this correlation is not significantly lower than the mean correlation coefficient of 0.57 determined for the full set of 465 livers by random permutation of 100 pairs of liver samples from the 465 liver study, indicating age does not have a significant effect of sex-biased liver gene expression. In addition, Pearson correlation coefficients = 0.58 and 0.59, respectively, were determined when comparing log2 male/female ratios of the 224 liver dataset to each of the two age-based subsets of the 465 liver dataset.

### Comparison to sex-biased genes in mouse and rat liver and analysis of non-synonymous versus synonymous substitution rates (dN/dS)

Orthologous gene pairs obtained from the mammalian orthology section of the Mouse Genome Informatics website, at http://www.informatics.jax.org/orthology.shtml, were used to identify mouse and rat orthologs of the human sex-biased genes that show sex-dependent expression in mouse or rat liver based on our earlier microarray studies; those studies used an Agilent mouse [27], [28] and an Agilent rat microarray platform [14], and a mouse microarray platform developed for Merck, Inc. [15], [34]. All non-duplicated genes that met the criteria of |fold change|>1.15 in combination with either *p*<0.005 (mouse Agilent platform) or *p*<0.05 (mouse Merck platform and rat Agilent platform) in at least one of the earlier studies were selected. The impact of hypophysectomy on the expression of mouse and rat liver sex-biased genes was based on our published data [14], [28]. The program codeml implemented in the PAML software package [35] was used to calculate the dN/dS ratio for human-mouse or human-rat orthologous gene pairs using the maximum-likelihood method [36]. When several gene accessions mapped to a given gene symbol, ratios of all accessions were calculated and the median dN/dS ratio was used. Non-sex-biased genes expressed in liver were identified based on these criteria: microarray signal intensity >100 in human liver; and |fold-change|<1.01 in both human and mouse (or rat) expression microarrays [14], [28]. 800 and 701 non-sex-biased human-mouse and human-rat orthologous were identified. Permutation testing was used to evaluate the difference between two medians. Specifically, we randomly selected two orthologous gene sets (each containing the same number of genes as the sets to be tested) and calculated the difference of the median, repeating the permutation 10,000 times. The number of times that the difference of the randomly selected group was higher than the observed difference was recorded as Nd. The permutation *p*-value was defined as Nd/10,000.

## Results

### Genes showing sex-biased expression in human liver

Human liver RNA was isolated from 112 male and 112 female livers, from which 16 male liver RNA pools and 16 female liver RNA pools were prepared and analyzed on two-color microarrays. 1,249 genes (transcripts) showing sex differences (sex bias) in expression were identified based on a combination of three criteria: mean |fold change| between male and female liver >1.15, *p*-value<0.005, and composite array score ≥14, with an apparent FDR<1% (see Methods). 873 of the 1,249 genes (70%) were expressed at a higher level in female liver and 376 genes (30%) were expressed at a higher level in male liver (Table S2A).

**Figure 1:**
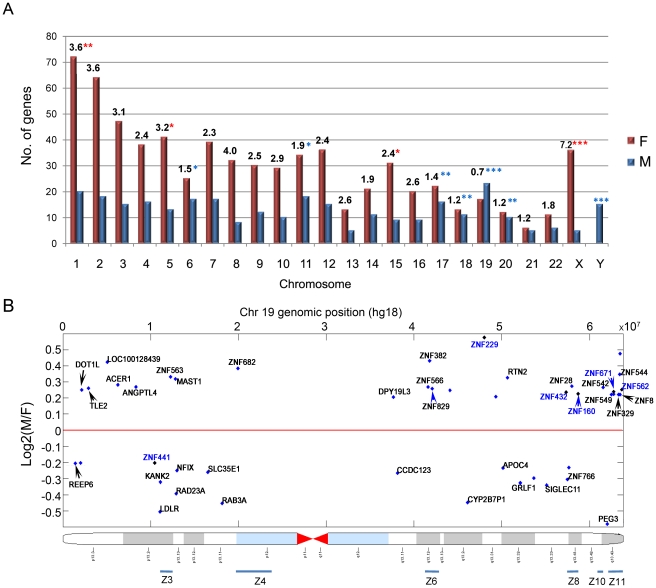
Chromosomal distribution of male-biased and female-biased genes identified in human liver. (A) Number of male- and female-biased genes on each chromosome, based on the criteria |fold change|≥1.15 and composite array score ≥14. Numbers at the top of each bar indicate the ratio of the number of female-biased genes to male-biased genes on each chromosome. Asterisks indicate the significance of the sex ratio based on Chi-square tests (*p<0.05; **p<0.01; ***p<0.001; red asterisks indicate significant enrichment of female-biased genes and blue asterisks indicate significant enrichment of male-biased genes). (B) Log2 male/female expression ratios for sex-biased genes on chromosome 19 vs. chromosomal location, based on genome release hg18. Blue bars at the bottom mark six previously defined *ZNF* gene clusters [40] that contain 13 sex-biased *ZNFs* in human liver; these *ZNF* genes show an enrichment score of ∼2.0 relative to the total number of *ZNF* genes on chromosome 19. An additional 6 sex-biased *ZNF* genes with a composite array score of 13 (Table S2C) are included, and marked with gene names shown in blue.

Analysis of the chromosomal distribution of genes showing sex-biased expression (Figure 1A; also see Figure S1 and Figure S2) revealed the highest male/female ratio for Y-chromosome genes, consistent with the sex assignments of the livers. The expressed Y-chromosome genes include *USP9Y*, a deubiquitination enzyme required for spermatogenesis, non-coding RNA transcripts such as *TTTY15*, and the JMJC domain histone demethylases *UTY and KDM5D*. 36 female-biased genes and 5 male-biased genes were found on the X-chromosome (Table 1, Table S2A and Figure S1B). These genes include zinc finger proteins (*ZFX*, *KDM5C*, *PHF6*, *MBNL3*, *ZMAT1*), transmembrane proteins (*IL1RAPL1, STS, EDA, GPR82, GJB1, PGRMC1*), and JMJC domain histone demethylases (*KDM6A, KDM5C*).

**Table 1:** Sex-biased genes on sex chromosomes (fold-change>|1.15| and composite array score ≥14).

Gene Symbol	Gene Accession	Fold-Change (F<0; M>0)	Gene Symbol	Gene Accession	Fold-Change (F<0; M>0)
**X-chromosome**					
*XIST*	NR_001564	−24.12	*MBNL3*	NM_133486	−1.29
*FRMD7*	NM_194277	−2.53	*GPR82*	NM_080817	−1.25
*ZFX*	NM_003410	−1.89	*SMC1A*	NM_006306	−1.25
*IL1RAPL1*	AJ243874	−1.86	*RIBC1*	NM_001031745	−1.24
*PNPLA4*	NM_004650	−1.71	*-*	ENST_00000436419	−1.22
*KDM6A*	NM_021140	−1.66	*TTC3L*	NR_030737	−1.21
*HDHD1A*	NM_012080	−1.62	*CXorf15*	NM_018360	−1.21
*GYG2*	NM_003918	−1.57	*LOC644538*	NM_001163438	−1.20
*STS*	NM_000351	−1.53	*FMR1*	NM_002024	−1.19
*ENOX2*	NM_182314	−1.48	*ZMAT1*	NM_032441	−1.19
*EIF1AX*	NM_001412	−1.46	*OPHN1*	NM_002547	−1.18
*CHM*	NM_000390	−1.43	*PGRMC1*	NM_006667	−1.17
*EDA*	NM_001399	−1.42	*DDX3X*	NM_001356	−1.16
*KDM5C*	NM_004187	−1.41	*GJB1*	NM_001097642	−1.16
*MUM1L1*	NM_152423	−1.38	*STAG2*	NM_006603	−1.15
*-*	AK022479	−1.37	*NXF3*	NM_022052	1.19
*CXorf38*	NM_144970	−1.35	*-*	AK124653	1.29
*MAP7D3*	NM_024597	−1.34	*-*	AK123627	1.30
*VCX2*	NM_016378	−1.33	*TAF7L*	NM_024885	1.42
*PHF6*	NM_032458	−1.32	*COL4A5*	NM_033381	1.52
*NCRNA00183*	NR_024582	−1.31			
**Y-chromosome**					
*USP9Y*	NM_004654	105.11	*CYorf15B*	BC035312	29.10
*ZFY*	NM_003411	58.63	*UTY*	NM_182660	23.68
*TTTY15*	NR_001545	35.53	*NLGN4Y*	NR_028319	19.66
*EIF1AY*	NM_004681	35.11	*NCRNA00185*	NR_001544	19.01
*CYorf15A*	NM_001005852	32.75	*KDM5D*	NM_004653	14.19

Fold-change indicates male/female expression value (positive values, for male-biased genes) and their negative inverse (negative values, for female-biased genes).Y-chromosome genes listed are those with an expression ratio >10.

### Partial escape from X-chromosome inactivation in human liver

Female-biased genes were enriched on the X-chromosome (*p*<0.001), which may reflect incomplete X-chromosome dosage compensation, whereby one X-chromosome is generally silenced (inactivated) in female cells [7], [8], [9]. *XIST*, an X-linked non-coding RNA gene that is a major effector of X-inactivation [37], showed the highest female/male expression ratio in our microarrays (Table 1). In a study using mouse/human hybrid cell lines that retain an inactive human X-chromosome, ∼15% of X-linked genes were found to be expressed from both X-chromosomes (i.e., escape or partially escape X-inactivation) [8], whereas only ∼5% of X-linked genes had this property in a study based on a panel of human lymphoblastoid cell lines [7]. Presently, we found that 4–6% of X-chromosome genes showed female-biased expression in liver (36, 44, and 50 X-linked genes, when assessed at composite array scores ≥14, ≥13, and ≥12, respectively; Figure 1A, Figure S2 and Table S2A, B). Furthermore, 10 of the 15 X-chromosome genes that showed consistent escape from X-inactivation in the human lymphoblastoid cell line study [7] showed consistent female-biased expression in human liver (composite array score = 16; Table 2), suggesting they escape X-inactivation in human liver as well. Indeed, all but two of the X-chromosome genes (*PGRMC1, PHF6*; Table 1) that showed female-biased expression in human liver also show female-bias in human muscle [38], supporting the conclusion that the female-biased expression of these genes reflects (partial) escape from X-inactivation, rather than hormone-based sex-bias or other mechanisms. In another study, 13 of 393 X-linked genes expressed in a female mouse kidney cell line escaped X-inactivation [39]. 11 of these 13 genes have corresponding human transcripts, 5 of which also showed female-biased expression in liver in all 16 arrays (*XIST, KDM6A, DDX3X, KDM5C, CXORF38*), suggesting they also escape X-inactivation in human liver.

**Table 2:** Comparison to genes that escape X-inactivation.

Gene Symbol	Alternate gene names	Fold-change	Microarray*p*-value	Composite array score	Agreement score of 4 populations (*p*<0.05)	Agreement score of 9 hybrids
*ZFX*		−1.89	0.00E+00	16	4	9/9
*PNPLA4*		−1.71	0.00E+00	16	4	9/9
*KDM6A*	*UTX*	−1.66	0.00E+00	16	4	9/9
*HDHD1A*		−1.62	0.00E+00	16	4	8/9
RPS4X *		−1.48	0.00E+00	16	4	9/9
*EIF1AX*		−1.46	0.00E+00	16	4	9/9
*KDM5C*	*JARID1C*	−1.41	0.00E+00	16	4	9/9
ZRSR2 *		−1.18	1.52E−21	16	4	N/A
*FUNDC1*		−1.11	7.28E−17	16	4	8/9
*DDX3X*		−1.16	1.18E−14	16^a^	4	9/9
*UBA1*	*UBE1*	−1.12	1.11E−10	14	4	9/9
*EIF2S3*		−1.17	1.28E−07	13	4	9/9
*USP9X*		−1.12	7.20E−05	12	4	9/9
*PRKX*		−1.07	NS	13	4	7/9
*CDK16*	*PCTK1*	1.01	NS	9	4	7/7

Listed are the top 15 genes that account for almost all of the differences in gene expression between males and females in lymphoblastoid cell lines [7]. Fold-change indicates magnitude and direction of sex-bias in expression, as in Table 1. The last two columns represent the occurrences of escape from X-inactivation for the indicated gene in a lymphoblastoid cell line study across 4 populations [7] and in a fibroblast cell hybrid study carried out in 9 hybrids [8]. NS, not significant. Two of the genes listed here (*) are not listed in Table 1 because their microarray probes have more than one hit in the human genome.

a - A second microarray probe for *DDX3X* exhibited a composite array score of 15 (Table S2A).

### Enrichment of male-biased genes on chromosome 19

Female-biased genes predominate on all autosomes except chromosome 19, where male-biased genes are significantly enriched (Figure 1A and Table 3; *p* = 2.7E-15 when compared to the overall distribution of sex-biased genes across the genome; see Methods). This same pattern characterized sex-biased genes identified at two lower levels of significance (composite array score >13 and composite array score >12, combined with |fold change|>1.15 and *p*-value<0.005; see Methods) (Figure S2). 16 of the 40 sex-biased genes on chromosome 19 are associated with transcription, and 13 are ZNF (zinc finger protein) transcription factors that map to six established *ZNF* clusters (Figure 1B) [40]. An additional 6 *ZNF* genes on chromosome 19 show sex-biased expression at a lower stringency (composite array score ≥13; Table S2C), and overall, 16 of the 19 sex-biased *ZNF* genes on chromosome 19 show higher expression in males.

**Table 3:** Sex-biased RefSeq genes on chromosome 19 (fold-change> |1.15| and composite array score ≥14).

GeneSymbol*	GeneAccession	Fold-Change	Sex-specificity	Gene Symbol	GeneAccession	Fold-Change	Sex-specificity
***ZNF382***	NM_032825	1.35	M	*REEP6*	NM_138393	−1.15	F
*LOC100128439*	BC032415	1.34	M	*APOC4*	NM_001646	−1.18	F
***ZNF682***	NM_033196	1.31	M	*NFIX*	NM_002501	−1.19	F
***ZNF544***	NM_014480	1.27	M	*SLC35E1*	NM_024881	−1.20	F
***ZNF563***	NM_145276	1.26	M	*CCDC123*	NM_032816	−1.20	F
*RTN2*	NM_206901	1.25	M	***ZNF766***	NM_001010851	−1.24	F
*MAST1*	NM_014975	1.25	M	*KANK2*	NM_015493	−1.25	F
*ACER1*	NM_133492	1.22	M	*GRLF1*	NM_004491	−1.25	F
***ZNF28***	NM_006969	1.21	M	*SIGLEC11*	NM_052884	−1.27	F
*ANGPTL4*	NM_139314	1.21	M	*RAD23A*	NM_005053	−1.31	F
***ZNF566***	NM_032838	1.21	M	*CYP2B7P1*	AK307933	−1.36	F
***ZNF542***	NR_033418	1.20	M	*RAB3A*	NM_002866	−1.37	F
*TLE2*	NM_003260	1.20	M	*LDLR*	NM_000527	−1.42	F
***ZNF829***	NM_001171979	1.20	M	***PEG3***	NM_006210	−1.50	F
*DOT1L*	NM_032482	1.19	M				
***ZNF549***	NM_153263	1.17	M				
***ZNF329***	NM_024620	1.17	M				
***ZNF8***	NM_021089	1.17	M				
*DPY19L3*	NM_207325	1.15	M				

*Gene symbols shown in bold identify 13 *ZNF* genes; an additional 6 *ZNF* genes (5 male-biased) show sex biased expression when the composite array score threshold is relaxed to 13 (see text).

### Functional analysis of human hepatic sex-biased genes

**Figure 2:**
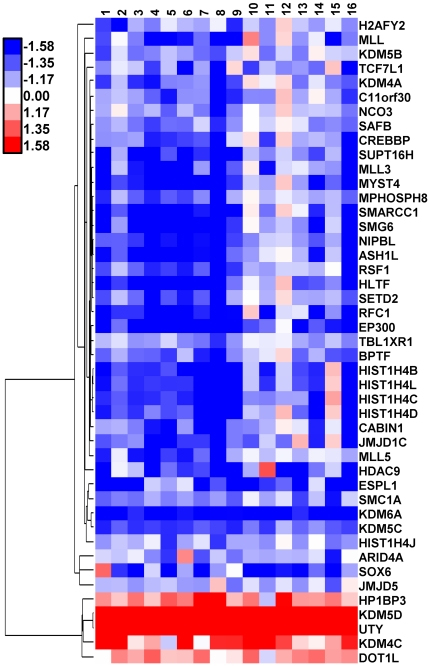
Heat map showing the male/female expression fold-change on each of 16 arrays for 45 sex-biased genes involved in chromatin remodeling and epigenetic modification. Blue indicates female-biased expression and red indicates male-biased expression, as shown in the linear color bar scale at top, left.

Functional clustering enrichment analysis [41], [42] revealed enrichment of female-biased genes in transcription and chromatin remodeling and epigenetic modification, pattern specification, cytoskeleton organization, cell junction and cell projection (Figure S3A), whereas male-biased genes are enriched in processes related to sexual reproduction (Figure S3B). Further details of the enriched functional clusters are provided in Table S3A. 277 of the 1,249 sex-biased genes encode proteins localized in nucleus (Table S3A) and 158 are involved in transcription regulation (Table S2D). Transcription factors showing sex-biased expression include 69 ZNFs, 9 homeo-box genes (female-biased *HOXB3, HOXD11, LHX2, ONECUT1, ONECUT2 and ZEB1*; and male-biased *CUX2*, *IRX3* and *PBX1*), and 6 female-biased nuclear receptors (*HNF4A, NR2C2, NR2F2, PGR, PPARA, RORA*). 45 sex-biased genes are significantly associated with chromatin organization and modification as determined by enrichment analysis (Figure 2, Table S2E, and Table S3A). These include 5 female-biased histone genes (*HIST1H4B, HIST1H4C, HIST1H4D, HIST1H4J, HIST1H4L*), 5 female-biased histone methyltransferase genes (*ASH1L, MLL, MLL3, MLL5, SETD2*), and one male-biased histone methyltransferase (*DOT1L*). Sex-biased genes containing JMJ domains with potential histone demethylase activity include *JMJD1C*, *JMJD5*, *KDM4A*, *KDM5B, KDM5C*, *KDM6A* (female-predominant) and *KDM4C*, *KDM5D* and *UTY* (male-predominant). Other sex-biased genes include a female-predominant histone deacetyltransferase (*HDAC9*) and several *histone* acetyltransferases (*CREBBP, EP300, MYST4, NCOA3*).

### Sex differences in lipid and drug metabolism

**Figure 3:**
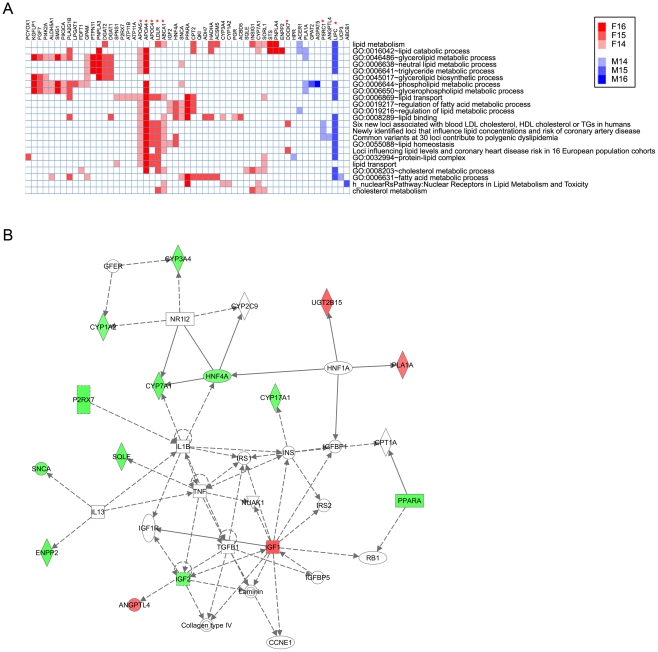
Association of sex-biased genes with lipid metabolism. (A) Heat map of 55 sex-biased genes associated with lipid metabolism with the 23 functional terms identified by DAVID analysis whose enrichment *p*-values are <1E-5. Each row represents a functional term and each column represents a gene, as marked at the top. Red, female-biased genes; blue, male-biased genes, with greater color intensity used to indicate genes with higher composite array scores, as shown in the color bar scale. Red asterisks mark 8 genes involved in polygenic dyslipidemia and cardiovascular disease as determined by GWAS [24], [45]. See Table S3B for a complete listing of 81 genes associated with lipid metabolism and the 57 corresponding functional terms. (B) Top network associated with sex-biased genes involved in lipid metabolism, as determined by Ingenuity Pathway Analysis. Other top networks are shown in Fig. S4.

Lipid metabolism was identified as the top molecular and cellular function significantly affected by the sex-biased genes (Table 4A and Table S4A). Lipid metabolic pathways, including fatty acid, cholesterol and triglyceride metabolism, encompassed 62 female-biased genes and 19 male-biased genes (Figure 3A, Table S2F and Table S3B). Top networks associated with these 81 genes include lipid metabolism, molecular transport and small molecule biochemistry (Figure 3B).

**Table 4:** Top biological functions and pathways affected by genes showing sex differences in human liver determined by Ingenuity Pathway analysis.

A. Top Biological Functions:		
1. Molecular and Cellular Functions		
Name	*p*-value range	No. sex-biased genes
Lipid Metabolism	1.03E-04–4.84E-02	42(81*)
Small Molecule Biochemistry	1.03E-04–4.84E-02	68
Molecular Transport	5.52E-04–2.47E-02	15
Cell Morphology	7.65E-04–4.84E-02	12
Gene Expression	1.30E-03–4.84E-02	94

2. Diseases and Disorders		
Name	*p*-value range	No. sex-biased genes
Cardiovascular Disease	2.56E-05–4.84E-02	164(185**)
Genetic Disorder	2.62E-05–5.00E-02	444
Endocrine System Disorders	4.51E-05–3.04E-04	171
Metabolic Disease	4.51E-05–4.84E-02	183
Neurological Disease	4.97E-04–3.58E-02	252

B. Top Canonical Pathways		
Name	*p*-value	No. sex-biased genes
TR/RXR Activation	5.12E-04	13
PTEN Signaling	7.16E-04	13
Cyanoamino Acid Metabolism	3.94E-03	5
Fructose and Mannose Metabolism	4.73E-03	8
Arachidonic Acid Metabolism	6.20E-03	13

C. Top Tox Functions		
Name	*p*-value	No. sex-biased genes
TR/RXR Activation	3.60E-04	13
Aryl Hydrocarbon Receptor (AhR) Signaling	6.24E-03	15
PXR/RXR Activation	1.66E-02	8
Cytochrome P450 Panel-Substrate is a Xenobiotic	1.86E-02	4
Positive Acute Phase Response Proteins	2.43E-02	5

*An additional 39 genes associated with lipid-related processes were identified based on functional terms collected by DAVID analysis.

**An additional 21 genes associated with cardiovascular disease were identified by querying against the Ingenuity Pathway disease database.

Cardiovascular disease is the most significant disease associated with sex-differential gene expression, and includes 185 sex-biased genes (Table 4A, Table S2G and Table S4A). Since dyslipidemia is a key risk factor for heart disease, we calculated the overlap and determined that 28 of the 81 sex-biased, lipid metabolism-related genes have previously been associated with cardiovascular disease (Table S2F). Moreover, 7 female-biased genes (*ABCA1, APOA4, APOA5, APOC4, DOCK7, HNF4A, LDLR*) and 3 male-biased genes (*ANGPTL4*, *LIPC*, *PSRC1*) are adjacent to a subset of the 30 loci previously associated with circulating concentrations of low density lipoprotein (LDL)-cholesterol, high density lipoprotein (HDL)-cholesterol and triglycerides and polygenic dyslipidemia by GWAS analysis [43], [44], with enrichment *p*-values ranging from 3E-9 to 5E-6 (Figure 3A, Table S2F and Table S3B). Furthermore, 8 of these 10 genes (all except *HNF4A* and *ANGPTL4*) are near 20 loci that influence lipid concentrations and risk of coronary artery disease [24], [45] (Figure 3A, Table S2F and Table S3B).

**Figure 4:**
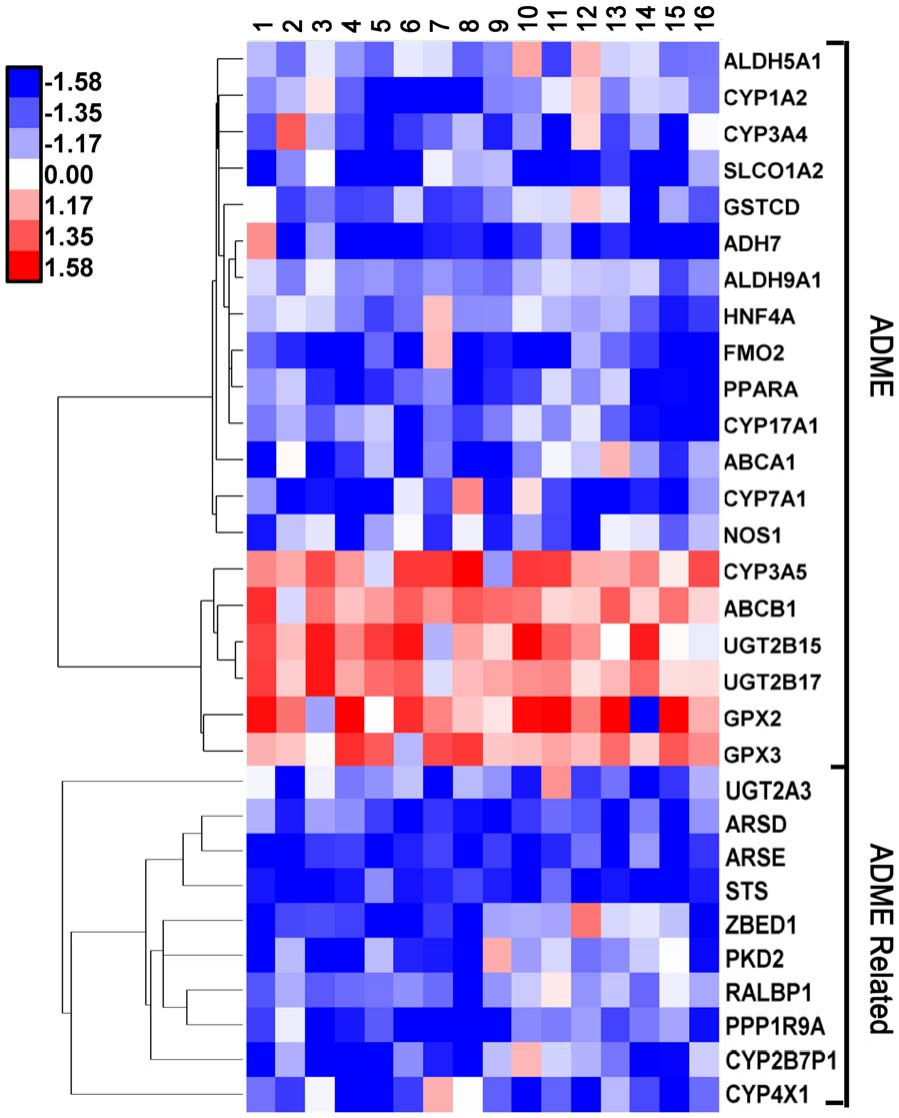
Heat map showing the male/female expression fold-change on each of 16 arrays for 20 ADME and 10 ADME-related genes that show sex-bias in human liver. Blue indicates female-biased expression and red indicates male-biased expression, as shown in the linear color bar scale at top, left.

The liver is the major site of drug clearance, and it expresses numerous drug-metabolizing enzymes belonging to the *CYP*, *UGT*, *GPX*, *ALDH* and other gene families. To fully evaluate the extent of sex-differences in drug-metabolizing enzyme expression and related processes, microarray data for 413 ADME and ADME-related genes were examined. We identified 30 ADME/ADME-related genes that show significant sex differences in expression at a composite array score ≥14 (Figure 4), and an additional 10 genes were identified when the composite array score was relaxed to 13 (Table S2H).

### Validation of key findings using a second liver cohort

To validate our microarray data, we examined an expression microarray dataset based of 465 individual human livers [33]. Of the 1,019 sex-biased RefSeq genes identified in our 224 liver dataset, 753 were represented in the 465 liver array dataset; these include 195 male-biased genes and 558 female-biased genes (see Methods). Comparison of the overall pattern of sex-biased genes between the two liver datasets by GSEA showed that the 195 male-biased genes identified in our study were significantly enriched in the male-biased gene set that we identified in the 465 liver study, with a normalized enrichment score (NES) = 2.8 and an enrichment *p*-value = 0. Similarly, the 558 female-biased genes identified in our study were significantly enriched in the corresponding female-biased genes from 465 dataset (NES = 2.5, *p*-value = 0). Furthermore, a Pearson correlation coefficient of 0.64 was determined for the 753 male/female log2-expression ratios of the two array datasets; this correlation exceeds the average correlation of 0.48 that we measured within the 465 liver dataset, based on permutation testing using random subsets of the 465 arrays (see Methods). Thus, our results show a good overall correlation with microarray results using a distinct microarray platform and an independent human liver cohort. Furthermore, 303 of the 753 common transcripts (40%) show a sex difference at *p*<0.05 in the 465 liver set, with 256 of the 303 genes showing the same sex-bias as seen in our 224 liver data set (84%; 76 male-biased genes and 180 female-biased genes), validating the sex-specificities of these genes. The finding of differences in sex bias for some individual genes in these two studies is not unexpected, given the differences in how the two studies were carried out (e.g., collection of fresh surgical samples at a single center in our study, vs. mostly post-mortem samples collected at three different centers and amplified at different points in time for the 465 liver dataset, different microarray platforms and probes used in each study, different tissue acquisition and storage protocols, criteria for RNA quality, etc.). Importantly, DAVID analysis of the 180 female-biased genes validated in the 465 liver study showed that lipid metabolism is a top functional cluster (Table S3D, S3E), the terms of which include all four GWAS-associated lipid metabolism/cardiovascular disease studies mentioned above [24], [43], [44], [45]. Taken together, these findings provide strong independent support for our major conclusion that human liver shows enrichment for sex-biased genes affecting lipid metabolism and cardiovascular disease.

### Comparison to genes that show sex-dependent expression in mouse and rat liver

Of the 1,249 genes showing sex-biased expression in human liver, 434 of 879 mouse orthologs showed sex-biased expression in mouse liver; similarly, 158 of 755 rat orthologs showed sex-biased expression in rat liver (Table S5A–S5C). Pituitary GH is the primary regulator of sex-dependent gene expression in mouse and rat liver [14], [28], including 75–77% of the sex-biased genes common to human and either mouse or rat liver, based on their responses to hypophysectomy (Table S5A–S5C). Gene Ontology analysis of these pituitary-dependent genes revealed top enriched functional terms associated with lipid metabolism, involving 41 genes in mouse and 33 genes in rat (Table S5D–S5E and and Table S2F), 21 of which are common to all three species (Table S2F). For those 87 genes that show sex-biased expression in all three species, 71 respond to hypophysectomy in mouse and 67 respond in rat (Table S5B and Table S5C). Lipid metabolism-associated terms are also the most significantly enriched in the 87 common sex-biased genes (Table S5F). Indeed, of the four sex-biased human liver genes directly linked to monogenic disorders of lipid metabolism, three show sex-biased gene expression in mouse liver (*Apoa5*, *Abca1*, *Lipc*), and in all three cases ablation of pituitary GH stimulation by hypophysectomy leads to their dysregulated expression in female mouse liver (Table S5B).

### Non-synonymous versus synonymous substitution rates in sex-biased genes

**Figure 5:**
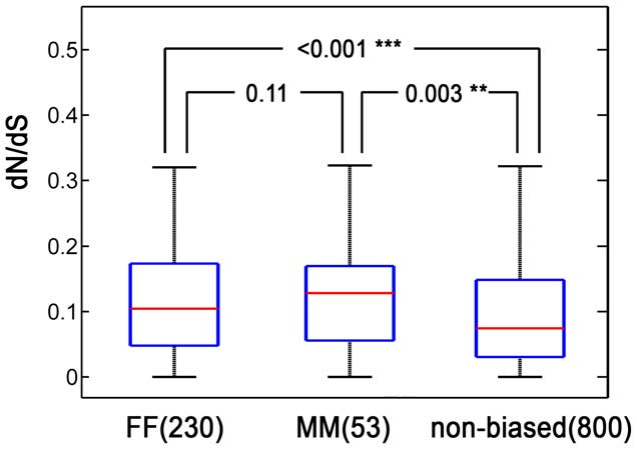
Non-synonymous versus synonymous substitutions in human and mouse sex-biased genes. Shown is a box plot of dN/dS ratios of common male-biased, female-biased and non-sex-biased genes between human and mouse. Numbers in parenthesis indicate the number of genes in each set. Common sex-biased genes that show consistent sex-bias in both human and mouse liver (MM and FF) had dN/dS ratios significantly higher than non-sex-biased genes (median of male-biased genes = 0.128, median of female-biased genes = 0.104, median of non-sex-biased genes = 0.074). Permutation *p*-values are indicated by **p<0.01 and ***p<0.001. The median dN/dS ratio for common male-biased genes was not significantly different than for female-biased genes (permutation *p*-value = 0.11).

Male-biased genes tend to evolve rapidly in protein-coding regions in both Drosophila [46] and primate brain [47]. To determine whether genes showing sex-biased expression in liver might also evolve rapidly, we compared the ratio of non-synonymous (amino acid-changing) to synonymous substitutions, dN/dS. Figure 5 shows that dN/dS ratios for human-mouse orthologs are significantly higher for both male-biased and female-biased genes than for non-sex-biased genes. In other systems, male-biased genes have a higher dN/dS ratio than female-biased genes [46], [47], however, in our data, the median dN/dS ratio for male-biased genes was not significantly higher than for female-biased genes (Figure 5). Similar patterns were seen for dN/dS ratios in sex-biased gene comparisons between rat and human liver but did not achieve statistical significance due to the small number of common sex-biased genes in these two species.

## Discussion

Sex differences in liver gene expression have been widely studied in rat and mouse models, where they have a major impact on hepatic physiology, inflammatory responses, diseased states, and the metabolism of steroids, drugs and environmental chemicals. However, little is known about sex-dependent gene expression in human liver, which could be of substantial biological and medical importance. Here, we report a comprehensive analysis of human liver sex differences based on a large panel of surgical tissue samples. More than 1,200 genes showing sex-biased expression are identified. Notably, several of the human hepatic sex-biased genes identified here have been previously associated with cardiovascular disease risk, with females characterized by a sex-biased expression profile consistent with their lower risk of coronary artery disease. Comparing our results with studies in the mouse, we find that half of the human-mouse orthologs also show sex-biased expression in mouse liver, although many genes reverse their sex bias. Those genes that show the same sex-bias in human and mouse liver are shown to evolve more rapidly than non-sex-biased genes. These findings provide novel insights into human hepatic sex differences important for processes such as drug metabolism and pharmacokinetics, and could help explain sex differential risk of coronary artery disease.

Sex differences in pathophysiology and disease risk characterize many tissues, including liver [48]. Large numbers of sex-dependent genes have been identified in mouse and rat liver, where male-female differences range from <2-fold to >1,000-fold [49]; however, previous global expression studies in human liver [19] and other tissues [38], [47], [50], [51] have been very limited in scope and lack sufficient statistical power to identify large numbers of sex-biased genes. The present study addressed this problem using a large panel of human livers and a pooling strategy that gives high statistical power, which enabled us to quantify sex differences in expression as low as 15%. Such differences can be biologically or medically relevant, even though they are small, in particular when multiple genes within a pathway are affected [52]. Using this approach, we identified many human hepatic genes that show sex-biased expression, affecting a broad range of biological processes important for human physiology and homeostasis, including lipid and drug metabolism. Key findings were validated by comparison to results reported for a 465 liver dataset [33], where an analysis of sex differences had not previously been carried out.

### Male-biased zinc finger clusters on chromosome 19

We identified 19 sex-dependent *ZNF* genes on chromosome 19, 16 of which showed male-biased expression and all but one of which map to 6 of 11 previously defined C2H2 type *ZNF* gene clusters on this chromosome (Fig. 1B; also see Table 3 and Table S2C) [40]. Notably, 15 of the 16 male-biased *ZNFs* contain a KRAB domain, which confers transcription repression [53], suggesting these *ZNFs* might target other liver-expressed genes and thereby contribute to female-biased gene expression. One of the female-biased *ZNF* genes, *PEG3*, is maternally imprinted, i.e., only the paternal allele is expressed [54], and is involved in signaling pathways regulated by NFKB, p53, tumor necrosis factor, and BAX [55]. *PEG3* DNA methylation is controlled by the transcription factor YY1 [56], which also showed female bias (*p* = 3.52E-11, composite array score = 15 and female/male ratio = 1.14). It will be interesting to determine whether sex differences characterize the epigenetic modifications surrounding the clusters of male-biased *ZNFs* on chromosome 19, in particular the six genes in *ZNF* cluster 11 (Table S2D).

### Epigenetics and sex differences in human liver

Epigenetic modifications play a critical role in sex differentiation, and recent evidence indicates a close association between gonadal sex steroids and both DNA and histone methylation [4], [10]. For example, CpG methylation and the histone modification pattern of the *Esr1* promoter is sexually dimorphic in mouse brain [57], [58], where sex steroid exposure can impart sex differences in DNA methylation [4]. KDM lysine demethylases may also contribute to sexual dimorphism *via* sex differences in their expression and/or intracellular distribution [4], [10], [11], [12]. Presently, we found that sex-biased genes were enriched in processes related to chromosome organization and modification, suggesting a role for genes such as the sex-linked JMJC domain histone demethylases (X-chromosome: *KDM5D*, *UTY*; Y-chromosome: *KDM5C*, *KDM6A*) [59], [60] in the establishment and/or maintenance of liver sexual dimorphism. Female-biased genes active in epigenetic modification, including *MLL* and *KDM6A*, may be key trans-regulators of *HOX* cluster gene expression [61], [62], [63], which is important for pattern formation during development. Notably, 22 genes associated with pattern specification processes showed female-biased expression, including two *HOX* genes (*HOXB3* and *HOXD11*) (Table S3A). Genes such as these could contribute to sex-biased differentiation of male and female liver during development.

### Sex-biased expression of hepatic drug-metabolizing enzymes

Sex differences in drug metabolism and pharmacokinetics can lead to sex differences in drug action and drug response, and have been related to the expression of key CYP enzymes of phase I (oxidative) drug metabolism [49]. Best documented is the female-biased expression of *CYP3A4* in human liver [17], [18], which was confirmed by our microarray analysis showing 29% higher *CYP3A4* expression in the female livers used in this study, a result that was validated by quantitative PCR using the same set of livers (30% female-biased expression; data not shown). Sex-differences in expression or activity have been reported for several other CYP enzymes (*CYPs 1A2*, *2B6*, *2C9*, *2D6*, *2C19* and *2E1*) but are not seen consistently and remain controversial [64]. Sex-biased expression has also been reported for certain human phase II drug-metabolizing enzymes of the *GST*, *UGT* and *ADH* families [49], [65], but can vary between ethnic groups [65]. Here, using a panel of liver samples from individuals of Western European descent, we identified 40 drug-metabolizing enzyme genes and other ADME or ADME-related genes that show sex-biased expression. These include *CYP1A2*, *CYP3A4* and *CYP7A1* showing female bias, and *CYP3A5*, *CYP27B1*, *APCS*, *PLA1A* and *UGT2B15* showing male bias (Figure 4 and Table S2H). In the case of *CYP1A2*, our finding of female-biased expression contradicts findings suggesting higher expression in males based on *in vivo* clearance rates of typical CYP1A2 substrates [64]. We also observed higher expression of *CYP3A5* in males, which contrasts to the female-biased expression of *CYP3A4*, although we cannot exclude a confounding effect of *CYP3A5* genetic polymorphisms [66].

Several nuclear receptors have been implicated as regulators of *CYPs* and other drug-metabolizing enzyme genes; these include HNF4A (NR2A1) and the xenobiotic-activated nuclear receptors CAR, PXR, and PPARA, which respond to a wide range of xenochemicals and induce the expression of *CYP2B*, *CYP3A*, and *CYP4A* and *CYP7A* genes, respectively [67], [68]. Steroid hormone-dependent responsiveness has been reported in rodent models for these receptors, as well as for AhR, the receptor/transcription factor that induces *CYP1* and other genes upon binding certain drugs and environmental chemicals [69]. *HNF4A* and *PPARA* both showed significant female-biased expression in human liver (Table S2A). *HNF4A* is a master regulator of gene expression in human liver [70], [71], and its female bias could contribute to the predominance of female-biased over male-biased genes (70% of the total) that we found in human liver. Consistently, several key nuclear receptor pathways are among the top pathways associated with sex-biased gene expression in human liver, including TR/RXR/LXR activation, AhR signaling, ER signaling, and PPAR signaling (Table 4B, Table 4C and Table S4B). Collectively, the observed sex differences in expression of drug metabolizing enzymes and other ADME genes may help explain clinical differences in drug response, including adverse drug reactions, which are frequently higher in females than in males [49].

### Association of sex dimorphism in lipid metabolism and heart disease

Dyslipidemia is a key risk factor in developing heart disease, whose lower incidence in women [22], [23] has been related to sex differences in lipid profiles [20], [21]. Thus, women typically have a more favorable lipid profile, with lower circulating levels of LDL (low density lipoprotein), higher levels of HDL, and lower triglyceride levels compared to men [20], [21]. These clinical observations are consistent with our finding that, of 8 sex-biased genes near loci associated with polygenic dyslipidemia and coronary heart disease [24], [45] (Table S2F), loss-of-function mutations in four genes result in monogenic disorders of lipid metabolism [72], [73], [74], [75], [76]. Strikingly, the sex-bias of these four genes (*LDLR*, *APOA5* and *ABCA1*, all more highly expressed in female liver; and *LIPC*, more highly expressed in male liver) is consistent with the more favorable lipid profile and lower cardiovascular disease risk profile of women. For example, familial hypercholesterolemia is induced by inherited defects in LDL receptor (*LDLR*), which disrupts hepatic control of circulating LDL-cholesterol [74]. Inherited *APOA5* deficiency is associated with severe hypertriglyceridemia [73] and, in another study [77], serum APOA5 concentrations were elevated in females compared to males, were negatively correlated with trigyceride concentrations in females, and were positively correlated to HDL-cholesterol levels in both males and females. Mutations in *ABCA1* have been associated with Tangier's disease and familial HDL deficiency [76], and individuals with high HDL-cholesterol levels have homozygous deficiencies of LIPC [75]. The increased expression of *LDLR*, *APOA5* and *ABCA1* that we observed in female liver, together with the lower expression of *LIPC*, can thus be expected to result in lower levels of LDL, lower triglycerides and higher levels of HDL in females, a lipid profile that predicts a lower risk of cardiovascular diseases. Other female-biased genes that we speculate contribute to the more favorable lipid metabolic profile of females include *CYP7A1*, encoding cholesterol 7α-hydroxylase, which catalyzes a key regulated step in the conversion of hepatic cholesterol to bile acids and is a target of bile acid sequestrants used to induce *CYP7A1* in the treatment of hypercholesterolemia [78], and PPARA, which is activated by hypolipidemic fibrate drugs [79]. *CYP7A1* and *PPARA* both showed ∼40% higher expression in female than male liver (Table S2A).

The favorable lipid profile of women has been ascribed to the protective effects of estrogen during a woman's reproductive years, although other factors, such as GH, which plays a major role in determining sex differences in rodent liver, could also be a factor. Clinical studies suggest that estrogen reduces LDL-cholesterol levels and increases HDL-cholesterol levels in post-menopausal women [80], [81]. Furthermore, estrogen deficiency may decrease rates of triglyceride metabolism by down-regulating transcription factors such as PPARA [82], a key regulator of lipid metabolism. The likely beneficial effects of the higher expression of *PPARA* seen here for female liver include increases in HDL levels, decreases in triglycerides via increased beta-oxidation, induction of *ABCA1*, increases in insulin sensitivity, and protection from atherosclerosis [83]. Notably, follicle-stimulating hormone (FSH) and luteinizing hormone (LH), which act synergistically in reproduction [84], are among the hub components in the top networks affected by the 1,249 sex-biased genes, as well as by the subset of 81 lipid-associated genes (Figure S4A, S4B, Table S4C). The relationship between FSH, LH and lipid metabolism suggested by these networks is consistent with a report that increased levels of FSH and LH in men with coronary artery disease are associated with increased levels of HDL-cholesterol, suggesting these hormones exert cardio-protective effects [85]. Another study reported, however, that elevated basal FSH was associated with unfavorable lipid levels (high LDL) and increased cardiovascular risk in normal cycling women [86].

### GH regulation of sex-biased gene: species similarities and species differences

A large majority of the sex differences in mouse and rat liver are regulated by GH [49], and correspondingly, a large fraction (75–77%) of the mouse and rat orthologs of sex-biased genes of human liver were characterized by pituitary hormone-dependence in mouse and/or rat liver; these include three of the four sex-biased human liver genes directly linked to monogenic disorders of lipid metabolism (*Apoa5*, *Abca1*, *Lipc*). These findings suggest that GH might also regulate the corresponding sex-biased genes in human liver, and by extension, the lipid metabolic processes and cardiovascular disease risks associated with these genes. Indeed, clinical studies indicate GH is an important determinant of lipid profiles in both healthy adults and GH-deficient patients [87], and clinically significant sex differences in GH responsiveness have been reported [88], [89]. Consistent with this proposal, two transcription factors implicated in the sex-dependent actions of GH in mouse and rat liver (CUX2, ONECUT2) [14], [28], also show sex-biased expression in human liver. GH can also exert sex-dependent effects on drug metabolism in humans [90], most likely through its effects on human hepatic CYP3A4 and other drug-metabolizing enzymes [49], several of which show strong GH-regulated hepatic sex differences when introduced into transgenic mice [91], [92]. The proposed role of GH in the regulation of sex-biased hepatic lipid and drug metabolism is an important area for further research.

Finally, species differences were apparent between human, mouse and rat, both with regards to the sex-specificity of individual genes (Table S5A) and the magnitude of sex differences (Table S5B and Table S5C). For example, of the 340 genes showing female-biased expression in human liver that also show sex-biased expression in mouse liver, 230 are more highly expressed in female mouse liver while 110 are more highly expressed in male mouse liver (Table S5A). At least some of these differences may be indicative of underlying species differences in associated physiological functions, such as the opposite sex-specificity of HDL-cholesterol levels in mice (male>female) [93] compared to humans [20]. Furthermore, the magnitude of sex differences in human liver is small (mostly <2-fold) compared to mouse and rat liver, where sex-differences in expression can range up to 1,000-fold.

In summary, this is the first comprehensive study of gene expression differences between sexes in human liver. More than 1,200 genes showing significant sex differences in expression and affecting diverse physiological functions were identified, with overall patterns and the key finding of sex differences in genes important for lipid metabolism and cardiovascular disease risk validated by analysis of an independent human liver cohort. These findings increase our understanding of sex differences in human liver at the molecular level and provide important insights into our understanding of clinical traits and drug responses. However, several limitations should be noted. First, the present analysis is based on liver samples from individuals of Western European descent, and needs to be validated for other cohorts, including livers representing other ethnic and racial groups. Second, the present study utilized fresh surgical specimens of non-tumorous tissue, primarily obtained from patients having primary liver tumors removed; however, there is no indication of sex-differences between such non-tumorous liver samples and livers obtained from non-tumor bearing donors, a supposition that is supported by our validation of key results using a second human liver cohort, primarily comprised of cancer-free post-mortem tissues. Third, the results presented are entirely based on microarray data, and it remains to be established to what extent the observed sex differences in gene expression will be indicative of sex differences at the protein level and at the level of biological activity. However, the striking consistency between our findings of sex-biased genes affecting lipid metabolism and cardiovascular disease risk and related clinical observations, discussed above, suggest that, at least in this area, our gene expression findings are functionally relevant. Finally, the present findings did not investigate sex-differences in gene expression and function that could arise from other, non-RNA-based mechanisms, such as translational regulation, protein stability and via post-translation modifications that alter biological function and activity. Further studies of sex-biased human hepatic genes at the genetic, regulatory and functional level can be expected to increase our understanding of their role in hepatic physiology and diseases states.

## Supporting Information

Figure S1 **Distribution of male- and female-biased genes on each chromosome.** (A) sex-biased genes are plotted against the male/female (M/F) log2 ratio. The length of the x-axis for each chromosome is proportional to the number of sex-biased genes. The three red lines represent male/female |fold-change| = 1.15, 0 and −1.15, respectively. (B) The log2 M/F expression ratios for sex-biased genes were plotted along the X-chromosome using coordinates based on hg18.
(TIF)

Figure S2 **Distribution of male- and female-biased genes on each chromosome based on less stringent levels of significance than shown in** **Fig. 1A** **.** Shown along the Y-axis are the numbers of male- and female-biased genes on each chromosome based on the combined criteria of |fold-change|>1.15 and either composite array score ≥13 (A) or composite array score ≥12 (B). Numbers at the top of each bar indicate the ratio of the number of female-biased genes to male-biased genes on each chromosome. Asterisks indicate the significance of the sex ratio based on Chi-square tests (*p<0.05; ** *p*<0.01; ***p<0.001; red asterisks indicate significant enrichment of female-biased genes and blue asterisks indicate significant enrichment of male-biased genes).
(TIF)

Figure S3 **Functional cluster enrichment analysis illustrating the biological functional terms enriched among sex-biased genes.** Shown are enriched functional terms associated with female-biased genes (A) or in male-biased genes (B). Statistically over-represented functional terms were determined by comparing the incidence of a functional term within the input gene list (observed, *blue bar*) to the incidence of that functional term among the entire human genes that have functional annotations collected by DAVID analysis (expected, *red bar*). Fisher's exact test was used to determine a *p*-value for each term.
(TIF)

Figure S4 **Top networks involving sex-biased genes identified by Ingenuity Pathway Analysis.** (A) Shows a top network of all sex-biased genes, which is associated with genetic disorder, reproductive system disease, cell-to-cell signaling and interaction. (B) Shows a top network of subset of sex-biased genes involved in lipid metabolism, which is associated with DNA replication, recombination, and repair, cell death and hepatic system disease. Green nodes indicate female-biased genes, and red nodes represent male-biased genes. Also see Fig. 3B.
(TIF)

Table S1 (A) Summary of patient data for livers included in this study, and (B) listing of 16 male and 16 female RNA pools used for microarray analysis.
(XLS)

Table S2 (A) Lists of all (1249) sex-biased genes based on criteria |fold-change|>1.15 and composite array score >14 including M/F expression ratio, *p*-value and composite array score. (B) An additional 1041 sex-dependent genes (411 male liver predominantly expressed genes and 630 female liver predominantly expressed genes) based on criteria composite array score > = 12, *p*<0.005 and |fold-change|>1.15. (C) 19 sex-dependent zinc fingers on chromosome 19 based on criteria: composite array score > = 13, *p*<0.005 and |fold-change|>1.15. (D) 158 sex-biased genes involved in transcription. (E) 45 sex-biased genes associated with chromatin organization and modification. (F) 81 sex-biased genes associated with lipid metabolism. (G) 185 sex-biased genes associated with cardiovascular disease. (H) 40 sex-biased ADME and ADME-related genes.
(XLS)

Table S3 (A) functional clusters enriched (enrichment score >1.5) for sex-biased genes. (B) 57 lipid-associated functional terms and 81 lipid-associated genes.
(XLS)

Table S4 Biological functions (A), canonical pathways (B) and networks (C) associated with human liver sex-biased genes identified by Ingenuity Pathway analysis.
(XLS)

Table S5 (A) Comparison of genes showing sex-biased expression in human, mouse and rat liver, and effect of hypophysectomy in mouse and rat. (B) List of 434 genes that show sex-biased expression in both human and mouse liver. (C) List of 158 genes that show sex-biased expression in both human and rat liver. (D–F) Listings of enriched functional clusters identified by DAVID (enrichment score >1.5) for genes that show sex-biased expression in human liver and either mouse liver (D) or rat liver (E) or mouse and rat liver (F), and where expression of the sex-biased mouse and rat genes is altered by hypophysectomy.
(XLS)
