# Supplementary figures and images for: Transcriptional Profiling of Human Liver Identifies Sex-Biased Genes Associated with Polygenic Dyslipidemia and Coronary Artery Disease
Source: PLoS One. 2011 Aug 12;6(8):e23506. doi: 10.1371/journal.pone.0023506 (PMC3155567; doi:10.1371/journal.pone.0023506)

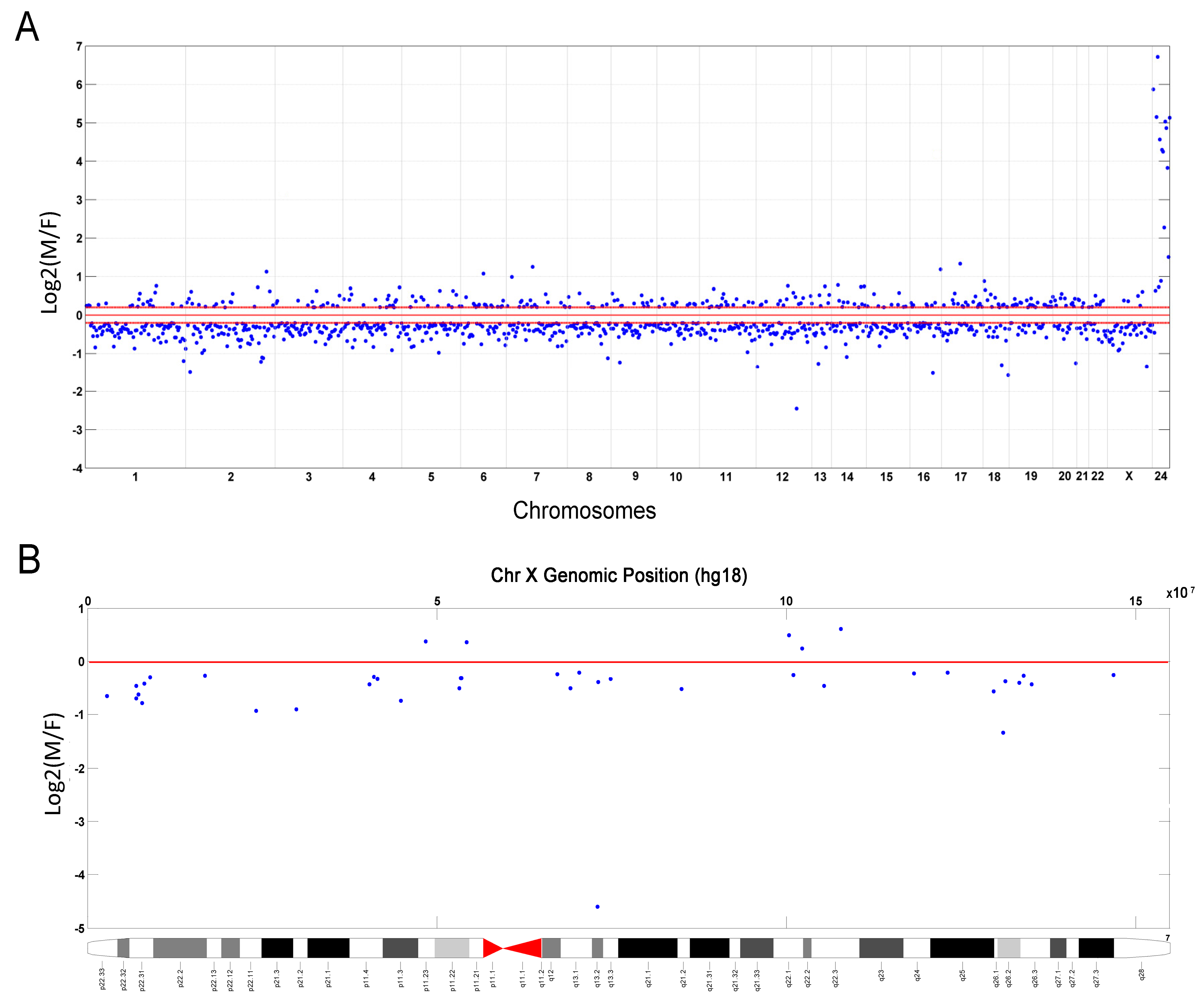

Supplement: Figure S1 — Distribution of male- and female-biased genes on each chromosome. (A) sex-biased genes are plotted against the male/female (M/F) log2 ratio. The length of the x-axis for each chromosome is proportional to the number of sex-biased genes. The three red lines represent male/female |fold-change| = 1.15, 0 and −1.15, respectively. (B) The log2 M/F expression ratios for sex-biased genes were plotted along the X-chromosome using coordinates based on hg18. (TIF) [file pone.0023506.s001.tif]

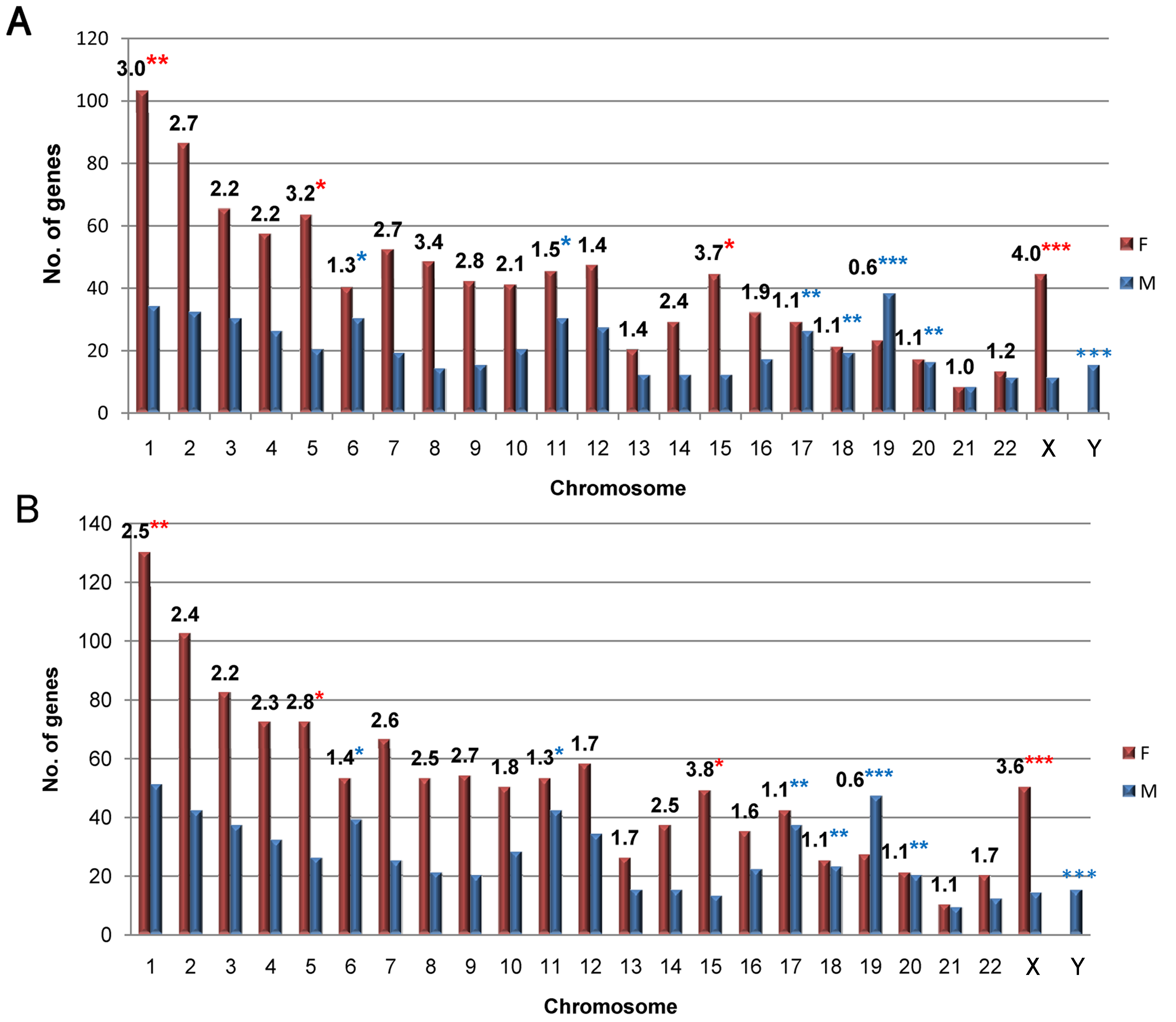

Supplement: Figure S2 — Distribution of male- and female-biased genes on each chromosome based on less stringent levels of significance than shown in Fig. 1A . Shown along the Y-axis are the numbers of male- and female-biased genes on each chromosome based on the combined criteria of |fold-change|>1.15 and either composite array score ≥13 (A) or composite array score ≥12 (B). Numbers at the top of each bar indicate the ratio of the number of female-biased genes to male-biased genes on each chromosome. Asterisks indicate the significance of the sex ratio based on Chi-square tests (*p<0.05; ** p<0.01; ***p<0.001; red asterisks indicate significant enrichment of female-biased genes and blue asterisks indicate significant enrichment of male-biased genes). (TIF) [file pone.0023506.s002.tif]

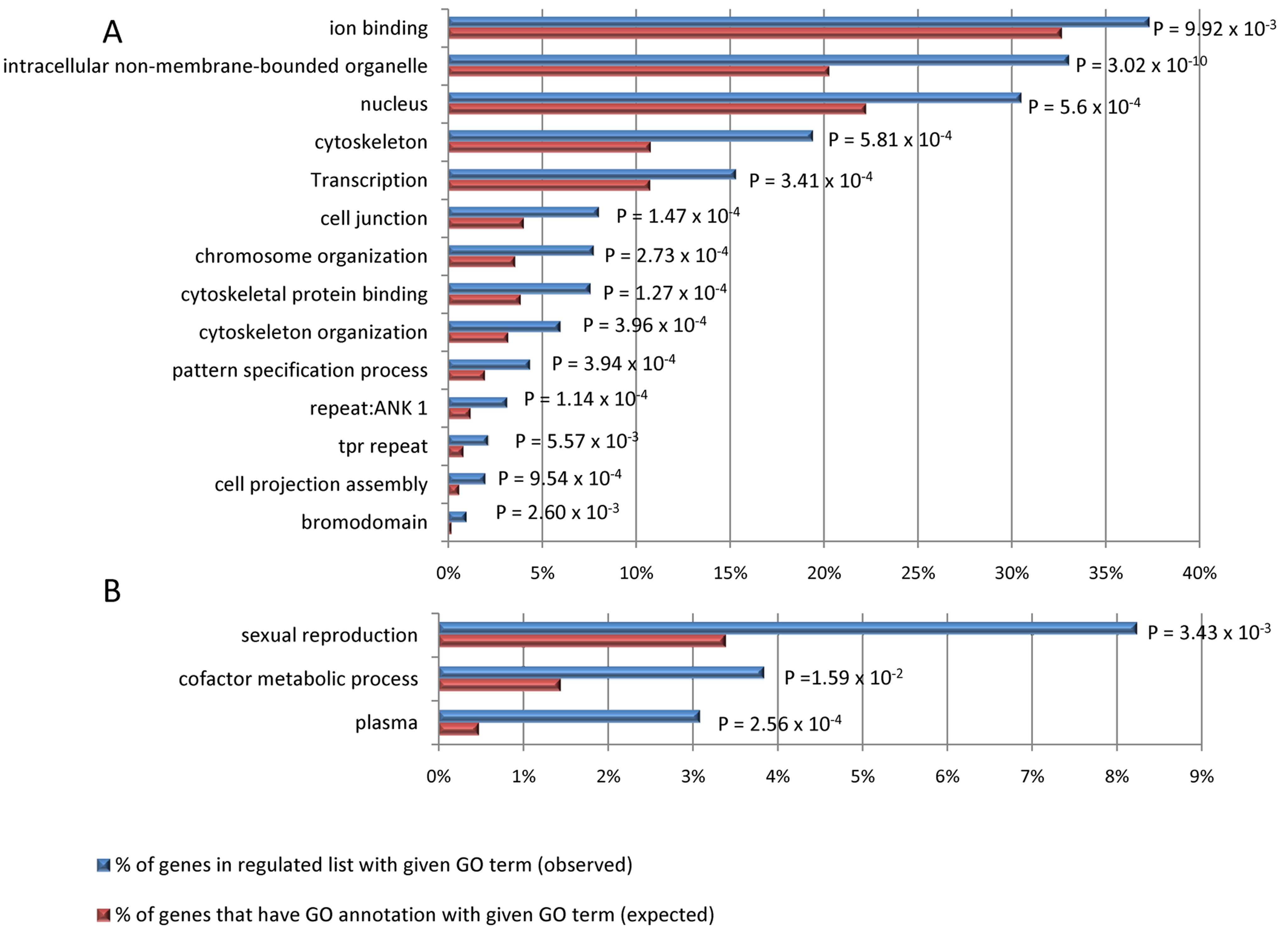

Supplement: Figure S3 — Functional cluster enrichment analysis illustrating the biological functional terms enriched among sex-biased genes. Shown are enriched functional terms associated with female-biased genes (A) or in male-biased genes (B). Statistically over-represented functional terms were determined by comparing the incidence of a functional term within the input gene list (observed, blue bar) to the incidence of that functional term among the entire human genes that have functional annotations collected by DAVID analysis (expected, red bar). Fisher's exact test was used to determine a p-value for each term. (TIF) [file pone.0023506.s003.tif]

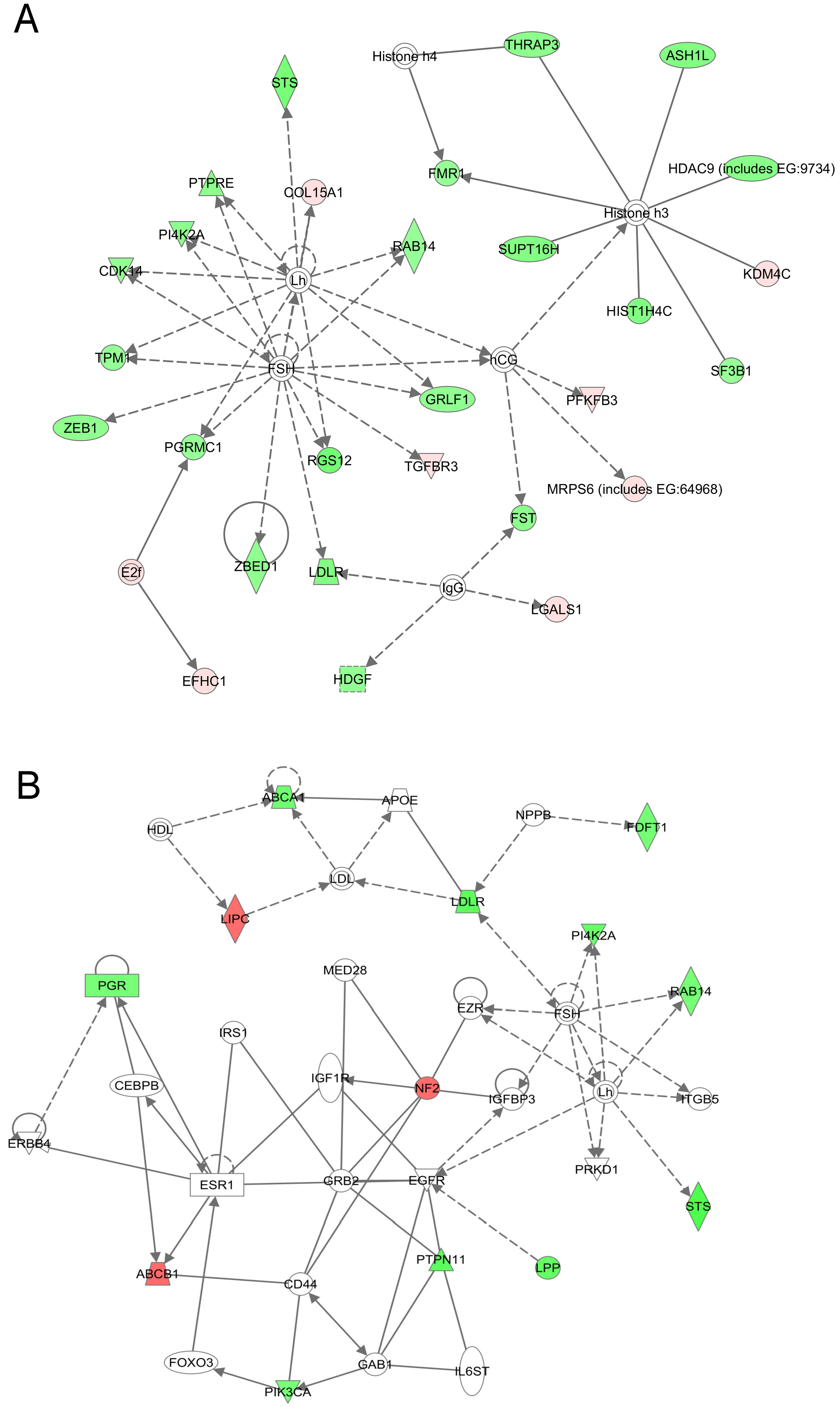

Supplement: Figure S4 — Top networks involving sex-biased genes identified by Ingenuity Pathway Analysis. (A) Shows a top network of all sex-biased genes, which is associated with genetic disorder, reproductive system disease, cell-to-cell signaling and interaction. (B) Shows a top network of subset of sex-biased genes involved in lipid metabolism, which is associated with DNA replication, recombination, and repair, cell death and hepatic system disease. Green nodes indicate female-biased genes, and red nodes represent male-biased genes. Also see Fig. 3B. (TIF) [file pone.0023506.s004.tif]
